# Overcompensation of herbivore reproduction through hyper‐suppression of plant defenses in response to competition

**DOI:** 10.1111/nph.14543

**Published:** 2017-04-07

**Authors:** Bernardus C. J. Schimmel, Livia M. S. Ataide, Rachid Chafi, Carlos A. Villarroel, Juan M. Alba, Robert C. Schuurink, Merijn R. Kant

**Affiliations:** ^1^ Department of Population BiologyInstitute for Biodiversity and Ecosystem DynamicsUniversity of AmsterdamPO Box 942401090 GEAmsterdamthe Netherlands; ^2^ Department of EntomologyFederal University of ViçosaCEP 36570‐000ViçosaBrazil; ^3^ Department of Plant PhysiologySwammerdam Institute for Life SciencesUniversity of AmsterdamPO Box 942151090 GEAmsterdamthe Netherlands

**Keywords:** competition, defense suppression, overcompensation, plant‐mediated interactions, spider mites, *Tetranychus evansi*, *Tetranychus urticae*, tomato (*Solanum lycopersicum*)

## Abstract

- Spider mites are destructive arthropod pests on many crops. The generalist herbivorous mite *Tetranychus urticae* induces defenses in tomato (*Solanum lycopersicum*) and this constrains its fitness. By contrast, the Solanaceae‐specialist *Tetranychus evansi* maintains a high reproductive performance by suppressing tomato defenses. *Tetranychus evansi* outcompetes *T. urticae* when infesting the same plant, but it is unknown whether this is facilitated by the defenses of the plant.
- We assessed the extent to which a secondary infestation by a competitor affects local plant defense responses (phytohormones and defense genes), mite gene expression and mite performance.
- We observed that *T. evansi* switches to hyper‐suppression of defenses after its tomato host is also invaded by its natural competitor *T. urticae*. Jasmonate (JA) and salicylate (SA) defenses were suppressed more strongly, albeit only locally at the feeding site of *T. evansi*, upon introduction of *T. urticae* to the infested leaflet. The hyper‐suppression of defenses coincided with increased expression of *T. evansi* genes coding for salivary defense‐suppressing effector proteins and was paralleled by an increased reproductive performance.
- Together, these observations suggest that *T. evansi* overcompensates its reproduction through hyper‐suppression of plant defenses in response to nearby competitors. We hypothesize that the competitor‐induced overcompensation promotes competitive population growth of *T. evansi* on tomato.

## Introduction

Arthropods and pathogens frequently attack plants in an attempt to gain access to nutrients. The extent to which plants can resist such attacks is determined by a plethora of mechanical and chemical defense mechanisms they acquired during the course of their evolution on the one hand and the degree to which their attackers can cope with these defenses on the other (Jones & Dangl, 2006; Howe & Jander, 2008). Following perception of the attacker, several signaling molecules, including phytohormones, orchestrate the plant's immune responses (Pieterse *et al*., 2012). In general, defenses against biotrophic pathogens and phloem‐feeding herbivores are regulated by the phytohormone salicylic acid (SA), while defenses against necrotrophic pathogens and chewing herbivores are regulated by jasmonates (JAs), in particular jasmonic acid‐isoleucine (JA‐Ile) (Glazebrook, 2005; Erb *et al*., 2012). Complex hormonal interaction networks enable tailoring of defenses, often via crosstalk, and modulate growth−defense tradeoffs (Pieterse *et al*., 2012; Huot *et al*., 2014).

Some plant defenses are induced not only in the attacked tissue, but also in systemic nonattacked tissues in order to prevent the parasite from simply evading the local defenses (Howe & Jander, 2008; Spoel & Dong, 2012). Furthermore, induced defenses may persist for some time after the initial attack has been averted. Considering that plants in nature often encounter multiple attackers, these attackers may interact with each other (also) via the plant. Such plant‐mediated interactions can impact on the fitness and behavior of organisms that are separated from each other in space and time and that belong to the same trophic level or to different levels (Ohgushi, 2005; Poelman & Dicke, 2014; Stam *et al*., 2014). In fact, plant‐mediated interactions have been established as a major factor influencing the performance of herbivorous arthropods (Kaplan & Denno, 2007) and, hence, the composition of plant‐associated arthropod communities (Van Zandt & Agrawal, 2004; Soler *et al*., 2005). The great majority of plant‐mediated interactions between herbivorous arthropods appear to be of an asymmetric and antagonistic nature, meaning that herbivory by one species negatively affects the performance of a second species via the induction of defenses (Kaplan & Denno, 2007; Poelman *et al*., 2008; Erb *et al*., 2009, 2011; Huang *et al*., 2014).

Counterintuitively, the induction of defenses by one herbivore species can also facilitate a second species on the same plant. Such plant‐mediated facilitation appears to be most common when herbivores from different feeding guilds feed from the same plant, and is thought to result from antagonistic crosstalk between the different defense signaling pathways that are induced by these herbivores (Soler *et al*., 2013). For example, phloem‐feeding aphids, which induce SA defenses, have been shown to improve the performance of leaf‐chewing caterpillars by interfering with JA defenses to which the caterpillars are susceptible (Kroes *et al*., 2015). Plant‐mediated facilitation does not, however, always or fully depend on antagonistic hormonal crosstalk, as herbivore‐induced changes in the plant's primary metabolism and reallocation of its resources may also play a role (Soler *et al*., 2013; Poelman & Dicke, 2014). In addition, the suppression of host plant defenses by a phytophagous organism may turn the attacked plant into a superior food source, that is, better than nonattacked plants, and may therefore attract, arrest and facilitate conspecifics (Sarmento *et al*., 2011a; Erwin *et al*., 2014), but also other phytophagous species (Glas *et al*., 2014; Li *et al*., 2014). Finally, plant‐mediated facilitation renders the plant more susceptible to plant parasites (known as induced susceptibility), resulting in overall more damage to the plant.

Here we have investigated the plant‐mediated interactions between the closely related and naturally competing spider mites *Tetranychus urticae* and *Tetranychus evansi*, which are herbivores from the same feeding guild. These spider mites (*c*. 0.5 mm in size) predominantly feed from mesophyll cells by piercing them with their stylet‐shaped mouthparts while avoiding damage to epidermal cells, after which they secrete saliva into the cell and suck up its contents (Helle & Sabelis, 1985; Bensoussan *et al*., 2016). *Tetranychus urticae* is a generalist found on over 1100 plant species, while *T. evansi* is considered a specialist on Solanaceae, and mites from the two species can co‐occur on the same host plant (Migeon *et al*., 2010; Navajas *et al*., 2013). Both mite species are a pest on tomato (*Solanum lycopersicum*), on which they cause significant economic losses (Saunyama & Knapp, 2004; Meck *et al*., 2013). On tomato plants, *T. urticae* induces a mixture of JA‐ and SA‐regulated defense responses (Kant *et al*., 2004; Martel *et al*., 2015) that constrains its performance (Li *et al*., 2002; Ament *et al*., 2004; Kant *et al*., 2008; Villarroel *et al*., 2016). By contrast, *T. evansi* was found to suppress JA and SA defenses (Sarmento *et al*., 2011a; Alba *et al*., 2015). This suppression was shown to act downstream of both JA and SA accumulation, and to be independent of JA–SA crosstalk (Alba *et al*., 2015). Furthermore, suppression could be attributed to secreted salivary proteins (effectors) (Jonckheere *et al*., 2016) of which at least two have been shown to suppress SA defenses (Villarroel *et al*., 2016).

Defense suppression is beneficial for *T. evansi* as it markedly increases its reproductive performance (Ataide *et al*., 2016) and reduces adult mortality (Sarmento *et al*., 2011a). However, defense‐inducing *T. urticae* females also produced significantly more eggs on leaflets previously (Sarmento *et al*., 2011b) or simultaneously (Alba *et al*., 2015) infested with *T. evansi* than on uninfested leaves or on leaves infested with conspecifics, respectively. This demonstrates plant‐mediated facilitation of *T. urticae* by *T. evansi*. Conversely, the reproductive performance of *T. evansi* was significantly reduced on leaflets previously attacked by *T. urticae* as compared with uninfested leaflets (Sarmento *et al*., 2011b). Together, these findings suggest that *T. urticae* outcompetes *T. evansi* when residing on the same plant. Yet, the reverse happens: in laboratory experiments *T. evansi* outcompetes *T. urticae* on tomato plants (Sarmento *et al*., 2011b), while also in the field, on natural vegetation, *T. evansi* was found to displace *T. urticae* (Ferragut *et al*., 2013; Azandémè‐Hounmalon *et al*., 2015).

Aiming to explain this paradox, we assessed the impact of *T. urticae* on the suppression of plant defenses by *T. evansi*. Previously, we showed that mixing *T. urticae* and *T. evansi* from the start on the same leaf results in intermediate suppression of defenses (Alba *et al*., 2015). In nature, however, mites usually infest plants sequentially (Ferragut *et al*., 2013; Glas *et al*., 2014). Therefore, here we first allowed *T. evansi* to establish its feeding site and introduced *T. urticae* to the adjacent leaf tissue 2 d later, and vice versa. Subsequently, we assessed (1) the spatial distribution of JA‐ and SA‐regulated defense responses at, and adjacent to, a mite's feeding site within infested leaflets; (2) the expression levels of effector‐encoding mite genes, and (3) the performance of the mites on the various feeding sites in the presence and absence of inter‐ or intraspecific competitors.

## Materials and Methods

### Plants

Tomato (*Solanum lycopersicum* L. cv Castlemart) and bean (*Phaseolus vulgaris* L. cv Speedy) plants were germinated and grown in a glasshouse (16 h : 8 h, 25°C : 18°C, day : night; 50–60% relative humidity (RH)). Experiments involving plants were carried out in a climate room (default settings: 25°C, 16 h : 8 h, light : dark, 60% RH, and 300 μmol m^−2^ s^−1^), to which plants were transferred 3 d in advance.

### Spider mites

We used spider mites from the *Tetranychus urticae* Koch Santpoort‐2 and *Tetranychus evansi* Baker & Pritchard Viçosa‐1 strains. The *T. urticae* Santpoort‐2 mites have been described before as inducers of tomato JA‐ and SA‐regulated defenses, to which they are also susceptible (Kant *et al*., 2008; Alba *et al*., 2015; Villarroel *et al*., 2016), while *T. evansi* Viçosa‐1 mites suppress these defenses (Sarmento *et al*., 2011a; Alba *et al*., 2015). Spider mites were reared on detached bean (for *T. urticae*) or tomato (for *T. evansi*) leaflets in a climate room. For all plant infestation experiments and mite performance assays, we used adult females of a similar age.

### Induction of plant defenses at a 4‐d‐old primary feeding site and in adjacent leaflet tissues including a 2‐d‐old secondary feeding site

#### General set‐up

To assess (1) the extent to which an infestation with either *T. urticae* or *T. evansi* mites influences the induced defense responses locally and systemically within a tomato leaflet, (2) how these defenses impact on the performance of heterospecific mites that are subsequently introduced to the same leaflet, and (3) how these secondary‐infestation mites alter the within‐leaflet defense responses, we divided tomato leaflets of intact plants into three sections (perpendicular to the midrib) and we successively infested two of them: during the first 2 d the middle section contained mites, and during the next 2 d the tip section also contained mites, after which phytohormone and gene expression analyses were performed on all three sections (Supporting Information Fig. S1).

#### Experimental procedure

We used one leaflet per plant: the second nonterminal leaflet of the second fully expanded leaf of 21‐d‐old plants. The adaxial surface of this leaflet was divided into a basal (i.e. at the petiolule side), middle and tip section, using a thin mite‐proof artificial barrier consisting of a mixture (50 : 50; v/v) of insect glue (Cola Entomológica Bio‐Controle, São Paulo, Brazil) and lanolin (Sigma‐Aldrich, St Louis, MO, USA). Two days later, the middle section was infested with either 25 *T. urticae* or 25 *T. evansi* mites. One group of plants (with the insect glue/lanolin barrier) remained uninfested and served as controls. For this primary infestation, we used 4–6‐d‐old adult females that originated from an ‘egg‐wave’ (Alba *et al*., 2015) made on detached bean leaflets and that had subsequently been habituated on tomato plants for 2 d. This habituation step was included to minimize possible effects of the previous diet (i.e. bean) on mite behavior, performance, and/or tomato responses (Storms, 1971). After 2 d of feeding by the 25 mites on the middle section, three 6–8‐d‐old adult *T. urticae* or *T. evansi* females (i.e. mites from the same egg wave as used for the primary infestation, but now habituated on tomato for 4 d) were introduced to the tip section of each leaflet (Fig. S1). For each primary infestation treatment, one‐third of the plants remained uninfested at the tip as controls. The experiment thus consisted of nine treatments: three primary infestation treatments (middle section of leaflet) and three secondary infestation treatments (tip section of leaflet). This experiment was performed in two blocks (experimental replicates) in time, in total with 45–56 plants per treatment (Table S1). Note that the basal leaflet section remained uninfested in all treatments.

#### Assessment of induced plant defenses

After a total of 4 d of infestation by the 25 mites on the middle section, from which 2 d with three hetero‐ or conspecifics on the tip section, the number of eggs and the number of live mites on the tip section were counted (nondestructively) using a stereo microscope (Leica MZ6; Leica Microsystems, Wetzlar, Germany). Subsequently, infested leaflets and corresponding uninfested control leaflets were excised without the petiolule, after which the basal, middle and tip sections were carefully cut out with a razor blade, thereby excluding leaf material covered with insect glue/lanolin (Fig. S1). The obtained leaflet parts were separated, flash‐frozen in liquid nitrogen and stored at −80°C until we extracted their phytohormones and isolated the RNA. As these leaflet parts were small, ten parts (i.e. either base, middle, or tip) obtained from ten plants were pooled to form one biological replicate to have a sufficient amount of leaf material to enable phytohormone extraction and RNA isolation from the same sample (Table S1).

### Transcript abundances of spider mite effector‐encoding genes on the primary feeding site in response to a secondary infestation of adjacent leaflet tissue

As the harvested middle section of mite‐infested leaflets contains both tomato tissue and spider mites (Fig. S1), the RNA isolated from these samples can be used for expression analyses of tomato and spider mite genes. Hence, we used the RNA isolated from leaflet middle sections that had been infested with mites for 4 d to assess the transcript abundances of *Te28* and *Te84* (for *T. evansi*) as well as *Tu28* and *Tu84* (for *T. urticae*), which hitherto are the only characterized spider mite genes that encode defense‐suppressing effectors (Villarroel *et al*., 2016).

### Performance of spider mites on the primary feeding site in response to a secondary infestation of adjacent leaflet tissue

#### General set‐up

To assess the extent to which the performance of spider mites from the primary infestation is affected by a secondary infestation of the leaflet tip section with heterospecifics, we repeated the infestation assay as described in the section ‘Induction of plant defenses at a 4‐d‐old primary feeding site and in adjacent leaflet tissues including a 2‐d‐old secondary feeding site’. After 4 d of infestation, we determined the performance of the 25 mites residing on the middle section of the leaflet.

#### Experimental procedure

For these experiments, the middle section of the leaflet was infested with either 25 *T. evansi* (65 plants) or 25 *T. urticae* (57 plants) females. Two days later, three *T. urticae* or three *T. evansi* females were introduced to the tip section of each leaflet. The tip section of one‐third of the plants remained uninfested as controls (Table S1). These experiments thus consisted of three treatments: one primary infestation treatment (the middle section of the leaflet) and three secondary infestation treatments (the tip section of the leaflet). The experiment with *T. evansi* as the primary infestation treatment was performed at a different moment in time from the experiment with *T. urticae* as the primary infestation treatment.

#### Spider mite performance

After 4 d of infestation, infested leaflets were excised and the number of live mites on the middle section was counted using a stereo microscope (Leica Microsystems). All mites were then removed from the middle section to expose the eggs. Each mite‐cleared leaflet was gently covered with a thin glass plate to flatten it out, after which it was photographed with a Nikon D2Xs DSLR camera (Nikon, Tokyo, Japan) equipped with an EL‐NIKKOR 50 mm f/2.8 lens (Nikon) to enable the *in silico* quantification of mite eggs using imagej (https://imagej.nih.gov/ij/).

### Isolation of phytohormones and analysis by means of liquid chromatography−tandem mass spectrometry (LC‐MS/MS)

Phytohormone analysis was performed following the procedure described by Alba *et al*. (2015) with minor modifications (Methods S1).

### Gene expression analysis by means of quantitative reverse transcriptase−polymerase chain reaction (qRT‐PCR)

Total RNA was isolated from tomato tissue (with or without mites) using the hot phenol method (Verwoerd *et al*., 1989). DNAse treatment, cDNA synthesis and qRT‐PCRs were performed following the procedures described by Alba *et al*. (2015) with minor modifications (Methods S2). We analyzed the transcript abundances of the tomato defense‐associated marker genes *12‐Oxo‐phytodienoic acid reductase 3* (*OPR3*), *Polyphenol‐oxidase‐D* (*PPO‐D*), *Jasmonate‐inducible protein 21* (*JIP‐21*), *Proteinase inhibitor IIc* (*PI‐IIc*), *Pathogenesis‐related protein 1a* (*PR‐1a*) and *Pathogenesis‐related protein P6* (*PR‐P6*), as well as the spider mite effector‐encoding genes *Te28*,* Te84*,* Tu28* and *Tu84*. Tomato *Actin* and spider mite *Ribosomal protein 49* (*RP49*) were used as reference genes for the respective template to normalize expression data across samples. Gene identifiers, primer sequences and references are listed in Table S2. To plot the relative gene expression, normalized expression (NE) values were scaled to the treatment with the lowest average NE.

### Statistical analysis

All the statistical analyses were performed with the software R v.3.1.3 (R Core Team, 2013) using either a generalized linear model (GLM) or a linear mixed‐effects model (LMER) in the lme4 package (Bates *et al*., 2015). Phytohormone concentration (per hormone), gene NE value (per gene), mite oviposition rate (per mite species), and survival (per mite species), respectively, were individually included in the model as the response variable (*y*), and treatment was included as the explanatory variable (*x*). The LMER included experimental replicate as a (random) factor in the model. Phytohormone and gene expression data were inspected for homogeneity of variances and normality of residuals, log‐ or sqrt‐transformed when necessary, and analyzed independently per leaflet section (i.e. base, middle and tip). Differences in the oviposition rate of spider mites were analyzed under a normal error distribution; differences in their survival were analyzed under a binomial error distribution (corrected for overdispersion). When significant differences were found, multiple comparisons were performed using Tukey contrasts in the multicomp package (Hothorn *et al*., 2008).

### Data availability

All raw data have been uploaded to FigShare (10.6084/m9.figshare.4702222) and are publicly available.

## Results

### Plant defenses at the spider mite feeding site and in adjacent uninfested leaflet tissues

First, as a benchmark we evaluated the local and systemic defense responses within tomato leaflets after an infestation with 25 *T. evansi* or 25 *T. urticae* adult females for 4 d. To do so, we restricted the mite infestation to one‐third of the tomato leaflet, namely, the middle section, and sampled the infested area as well as the uninfested adjacent tissues for phytohormone content and defense gene expression analyses (Fig. S1).

#### Phytohormone accumulation

**Figure 1:**
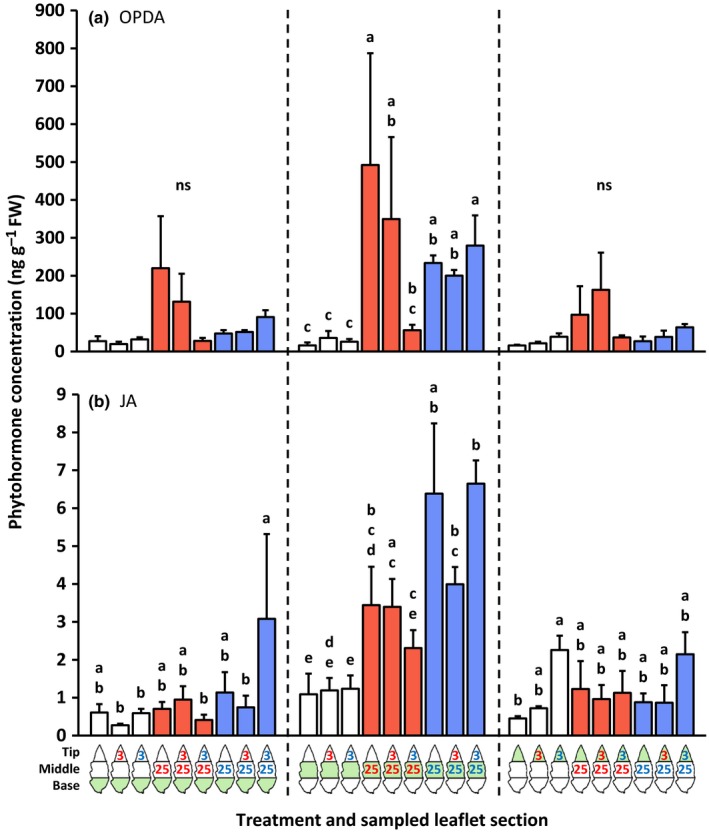
Amounts of 12‐oxo‐phytodienoic acid (OPDA) and jasmonic acid (JA) in basal, middle and tip sections of spider mite‐infested tomato (*Solanum lycopersicum*) leaflets. Using artificial barriers, leaflets of intact plants were divided into three sections: base, middle and tip. The middle section was infested with 25 *Tetranychus evansi* mites (red letters) or 25 *Tetranychus urticae* mites (blue letters), or remained uninfested as a control. After 2 d, the tip section was subjected to a secondary infestation with either three *T. evansi* mites or three *T. urticae* mites. Leaflets with uninfested tip sections were used as controls. Again 2 d later, leaflets were excised and leaflet sections were cut out for hormone extraction and RNA isolation (Supporting Information Fig. S1). The figure shows the average (+ SEM) amounts of (a) OPDA and (b) JA in each of the leaflet sections. The leaflet section that was sampled is indicated in green in the diagram below the bar graphs. Bars are colored according to the treatment of the middle section (primary infestation). Phytohormone data were statistically evaluated per leaflet section. Different letters above the bars indicate significant differences at a level of *P *≤* *0.05, after applying a linear mixed‐effects model followed by Tukey multiple comparisons. ns, not significant.

**Figure 2:**
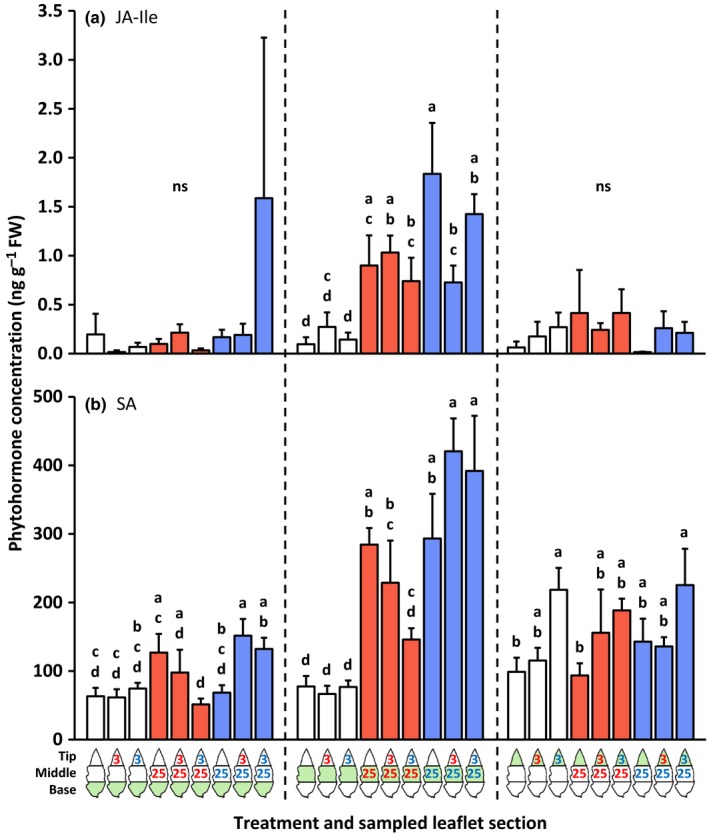
Amounts of jasmonic acid‐isoleucine (JA‐Ile) and salicylic acid (SA) in basal, middle and tip sections of spider mite‐infested tomato (*Solanum lycopersicum*) leaflets. Using artificial barriers, leaflets of intact plants were divided into three sections: base, middle and tip. The middle section was infested with 25 *Tetranychus evansi* mites (red letters) or 25 *Tetranychus urticae* mites (blue letters), or remained uninfested as a control. After 2 d, the tip section was subjected to a secondary infestation with either three *T. evansi* mites or three *T. urticae* mites. Leaflets with uninfested tip sections were used as controls. Again 2 d later, leaflets were excised and leaflet sections were cut out for hormone extraction and RNA isolation (Supporting Information Fig. S1). The figure shows the average (+ SEM) amounts of (a) JA‐Ile and (b) SA in each of the leaflet sections. The leaflet section that was sampled is indicated in green in the diagram below the bar graphs. Bars are colored according to the treatment of the middle section (primary infestation). Phytohormone data were statistically evaluated per leaflet section. Different letters above the bars indicate significant differences at a level of *P *≤* *0.05, after applying a linear mixed‐effects model followed by Tukey multiple comparisons. ns, not significant.

Compared with the uninfested control, mites from both species induced a significant accumulation of 12‐oxo‐phytodienoic acid (OPDA), JA, JA‐Ile and SA locally, that is, at their respective feeding sites, but not systemically, that is, in the uninfested adjacent leaflet parts (Figs 1, 2; the leftmost bar of each group of three bars with the same color). There were no statistically significant differences between the concentrations of these phytohormones at the *T. evansi* and *T. urticae* feeding sites.

#### Defense gene expression

**Figure 3:**
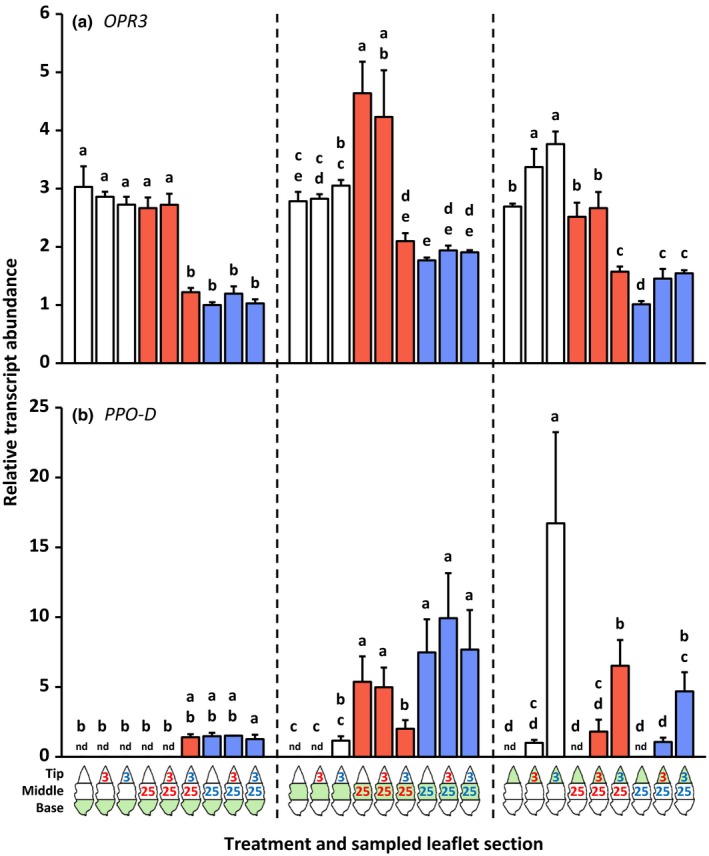
Relative transcript abundances of the wounding/jasmonic acid marker genes *12‐Oxo‐phytodienoic acid reductase 3* (*OPR3*) and *Polyphenol‐oxidase‐D* (*PPO‐D*) in basal, middle and tip sections of spider mite‐infested tomato (*Solanum lycopersicum*) leaflets. Using artificial barriers, leaflets of intact plants were divided into three sections: base, middle and tip. The middle section was infested with 25 *Tetranychus evansi* mites (red letters) or 25 *Tetranychus urticae* mites (blue letters), or remained uninfested as a control. After 2 d, the tip section was subjected to a secondary infestation with either three *T. evansi* mites or three *T. urticae* mites. Leaflets with uninfested tip sections were used as controls. Again 2 d later, leaflets were excised and leaflet sections were cut out for hormone extraction and RNA isolation (Supporting Information Fig. S1). The figure shows the average (+ SEM) normalized transcript abundances of (a) *OPR3* and (b) *PPO‐D* in each of the leaflet sections. The leaflet section that was sampled is indicated in green in the diagram below the bar graphs. Transcript abundances were normalized to *Actin* and then scaled to the overall lowest average value per gene panel. Bars are colored according to the treatment of the middle section (primary infestation). Gene expression data were statistically evaluated per leaflet section. Different letters above the bars indicate significant differences at a level of *P *≤* *0.05, after applying a linear mixed‐effects model followed by Tukey multiple comparisons. nd, not detected.

**Figure 4:**
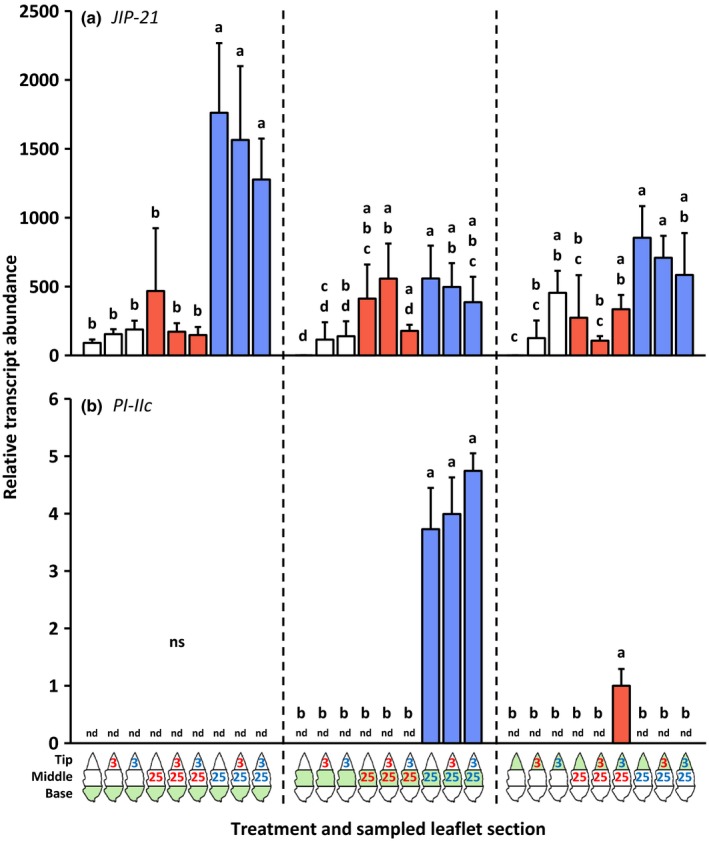
Relative transcript abundances of the jasmonic acid defense marker genes *Jasmonate‐inducible protein 21* (*JIP‐21*) and *Proteinase inhibitor IIc* (*PI‐IIc*) in basal, middle and tip sections of spider mite‐infested tomato (*Solanum lycopersicum*) leaflets. Using artificial barriers, leaflets of intact plants were divided into three sections: base, middle and tip. The middle section was infested with 25 *Tetranychus evansi* mites (red letters) or 25 *Tetranychus urticae* mites (blue letters), or remained uninfested as a control. After 2 d, the tip section was subjected to a secondary infestation with either three *T. evansi* mites or three *T. urticae* mites. Leaflets with uninfested tip sections were used as controls. Again 2 d later, leaflets were excised and leaflet sections were cut out for hormone extraction and RNA isolation (Supporting Information Fig. S1). The figure shows the average (+ SEM) normalized transcript abundances of (a) *JIP‐21* and (b) *PI‐IIc* in each of the leaflet sections. The leaflet section that was sampled is indicated in green in the diagram below the bar graphs. Transcript abundances were normalized to *Actin* and then scaled to the overall lowest average value per gene panel. Bars are colored according to the treatment of the middle section (primary infestation). Gene expression data were statistically evaluated per leaflet section. Different letters above the bars indicate significant differences at a level of *P *≤* *0.05, after applying a linear mixed‐effects model followed by Tukey multiple comparisons. nd, not detected; ns, not significant.

**Figure 5:**
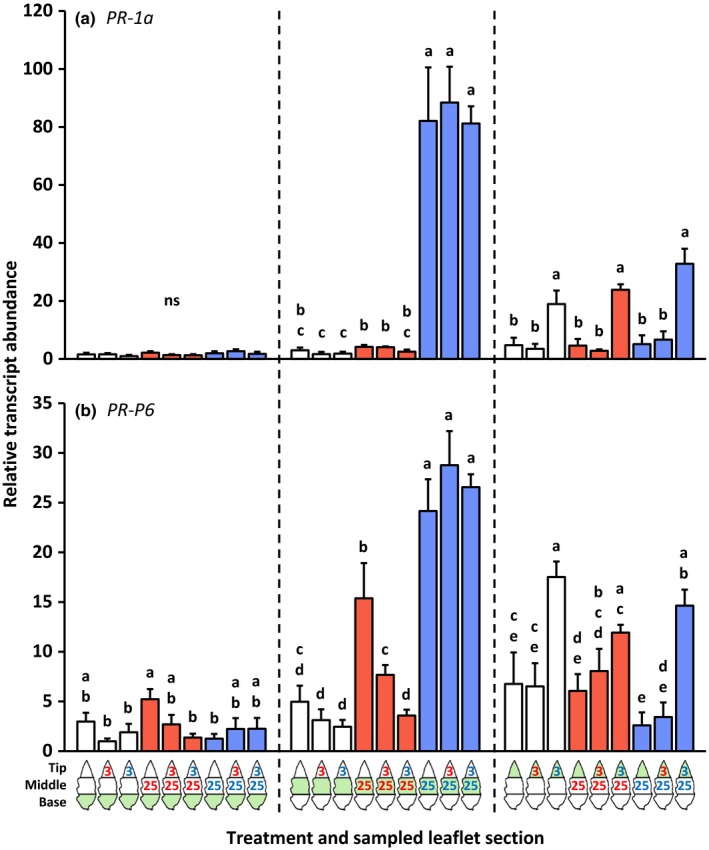
Relative transcript abundances of the salicylic acid defense marker genes *Pathogenesis‐related protein 1a* (*PR‐1a*) and *Pathogenesis‐related protein P6* (*PR‐P6*) in basal, middle and tip sections of spider mite‐infested tomato (*Solanum lycopersicum*) leaflets. Using artificial barriers, leaflets of intact plants were divided into three sections: base, middle and tip. The middle section was infested with 25 *Tetranychus evansi* mites (red letters) or 25 *Tetranychus urticae* mites (blue letters), or remained uninfested as a control. After 2 d, the tip section was subjected to a secondary infestation with either three *T. evansi* mites or three *T. urticae* mites. Leaflets with uninfested tip sections were used as controls. Again 2 d later, leaflets were excised and leaflet sections were cut out for hormone extraction and RNA isolation (Supporting Information Fig. S1). The figure shows the average (+ SEM) normalized transcript abundances of (a) *PR‐1a* and (b) *PR‐P6* in each of the leaflet sections. The leaflet section that was sampled is indicated in green in the diagram below the bar graphs. Transcript abundances were normalized to *Actin* and then scaled to the overall lowest average value per gene panel. Bars are colored according to the treatment of the middle section (primary infestation). Gene expression data were statistically evaluated per leaflet section. Different letters above the bars indicate significant differences at a level of *P *≤* *0.05, after applying a linear mixed‐effects model followed by Tukey multiple comparisons. ns, not significant.

The same leaf samples as used for the phytohormone analysis were also used to determine the transcript abundances of defense‐associated genes (Figs 3, 4, 5; the leftmost bar of each group of three bars with the same color). Locally, *PI‐IIc* transcripts were only detected after infestation with *T. urticae* (Fig. 4b), whereas *PR‐1a* (Fig. 5a) and *PR‐P6* (Fig. 5b) were up‐regulated significantly more strongly by *T. urticae* than by *T. evansi*. Systemically, the expression of these three genes was not up‐regulated after mite feeding. *OPR3* was locally significantly induced by *T. evansi* but not by *T. urticae* (Fig. 3a). Yet, in the leaf tissues adjacent to the *T. urticae*‐infested area, but not adjacent to the *T. evansi*‐infested area, *OPR3* was down‐regulated. Mites from the two species induced *PPO‐D* (Fig. 3b) and *JIP‐21* (Fig. 4a) at their feeding sites to similar levels. In addition, *JIP‐21* was highly up‐regulated in both uninfested leaf areas adjacent to the *T. urticae* feeding site.

### Plant defenses at the primary feeding site and in adjacent leaflet tissues upon a secondary infestation of the same leaflet

Second, we assessed how the within‐leaflet defense responses that are induced or suppressed by mites are altered by a secondary infestation of the same leaflet by introducing three hetero‐ or conspecific mites to the tip section 2 d after the initial (primary) infestation. Again, we analyzed defense responses in all three leaflet parts 4 d after infestation of the middle section (Fig. S1).

#### Phytohormone accumulation at the 2‐d‐old secondary feeding site (the tip part of the leaflet)

The leaflet tip accumulated more SA in response to *T. urticae*; this was most noticeable when the middle part was not infested (Fig. 2b). The SA concentration did not increase in the tip in response to *T. evansi*. For JA, a similar pattern was observed, although there was no induction by *T. urticae* in the tip when it was adjacent to a *T. evansi* feeding site (Fig. 1b). OPDA and JA‐Ile concentrations did not significantly increase in the tip sections in response to the secondary infestation (Figs 1a, 2a).

#### Phytohormone accumulation at the 4‐d‐old primary feeding site (the middle part of the leaflet)

Leaflets that were not infested in the middle but only at the tip (for 2 d) did not have significantly altered concentrations of OPDA, JA, JA‐Ile or SA in the middle (and basal) sections compared with uninfested controls. At the primary *T. urticae* feeding site, however, JA‐Ile concentrations decreased upon the secondary infestation with *T. evansi* (Fig. 2a). At the primary *T. evansi* feeding site, concentrations of OPDA (Fig. 1a) and SA (Fig. 2b) were significantly lower when the tip had been infested with *T. urticae* compared with when it had remained uninfested. We observed the same trend for JA and JA‐Ile, but these differences were not statistically significant (Figs 1b, 2a).

#### Defense gene expression at the 2‐d‐old secondary feeding site (the tip part of the leaflet)

The secondary infestation of the leaflet tip section with *T. urticae* resulted in the local up‐regulation of *PR‐1a* (Fig. 5a) and *PR‐P6* (Fig. 5b). The expression of these *PR* genes was not induced by *T. evansi*; hence, their expression patterns paralleled the SA concentrations. Compared with the control treatments, the secondary infestation with *T. urticae* also up‐regulated *PPO‐D* expression in the tip section (Fig. 3b). Although *PPO‐D* transcripts were merely detected in the tip part after it had been infested, transcript levels were significantly higher in response to *T. urticae* than in response to *T. evansi*. Notably, induction of *PPO‐D* in the *T. urticae*‐infested tip part was highest when the adjacent leaf area was uninfested. Similarly, *JIP‐21* expression in the tip part was only significantly induced upon local infestation with *T. urticae* and when the middle section was uninfested (Fig. 4a). For these treatments (i.e. middle section uninfested), the *PPO‐D* and *JIP‐21* expression patterns thus best matched JA concentrations. The secondary infestation of the tip with either *T. urticae* or *T. evansi* resulted in an increased abundance of *OPR3* transcripts when the middle section was infested with *T. urticae* (i.e. similar to when the middle section was uninfested), but in a decreased abundance of *OPR3* transcripts when *T. evansi*‐infested leaflets were subjected to a secondary infestation with *T. urticae* (Fig. 3a).

#### Defense gene expression at the 4‐d‐old primary feeding site (the middle part of the leaflet)

Compared with uninfested controls, leaflets that were only infested at the tip (for 2 d) did not have significantly altered transcript levels of *OPR3*,* PPO‐D*,* JIP‐21*,* PI‐IIc*,* PR‐1a* or *PR‐P6* in the middle (and basal) sections (Figs 3, 4, 5). Also at the primary *T. urticae* feeding sites we did not detect significant differences in the expression of these genes upon the secondary infestation, despite differences in the JA‐Ile concentration. By contrast, at the primary *T. evansi* feeding sites, the expression of *PR‐P6* was suppressed more strongly (i.e. hyper‐suppressed) upon the secondary infestation with either of the mite species (Fig. 5b). Strikingly, *PR‐P6* transcript levels were lowest after *T. urticae* had infested the adjacent tip section. Also, the transcript accumulation of *OPR3* (Fig. 3a) and *PPO‐D* (Fig. 3b) at *T. evansi*'s primary feeding site was suppressed upon the secondary infestation with *T. urticae*. Although differences were not statistically significant, the same trend was observed for *JIP‐21* (Fig. 4a) and *PR‐1a* (Fig. 5a), but not for *PI‐IIc*, because this gene was already fully suppressed by *T. evansi* (Fig. 4b).

#### Spider mite performance at the secondary feeding site

For spider mites, the highest food conversion is achieved by young, adult females (i.e. as used in our experiments), which at 25°C can produce up to 12 eggs d^−1^, equivalent to 60% of their body weight (Sabelis, 1981; Gotoh *et al*., 2010). Moreover, their peak oviposition rate and intrinsic rate of population increase are significantly correlated, such that the former can be used as an adequate proxy for the latter (Sabelis, 1991; Janssen & Sabelis, 1992). Before the harvest of the infested tomato leaflets for the phytohormone and gene expression analyses, we counted the number of eggs produced by the three mites on the tip section during the 2‐d (co‐)infestation period. The oviposition rates of the *T. evansi* and *T. urticae* females on the leaflet tip were similar and were not significantly influenced by the primary infestation treatment (Fig. S2). In addition, the survival of mites on the leaflet tip was not significantly affected by the primary infestation treatment either (for *T. evansi*: LMER; χ²_[2,4]_ = 1.25; *P* = 0.54; for *T. urticae*: LMER; χ²_[2,4]_ = 0.56; *P* = 0.76).

### Transcript abundances of spider mite effector‐encoding genes on the primary feeding site in response to a secondary infestation of adjacent leaflet tissue

**Figure 6:**
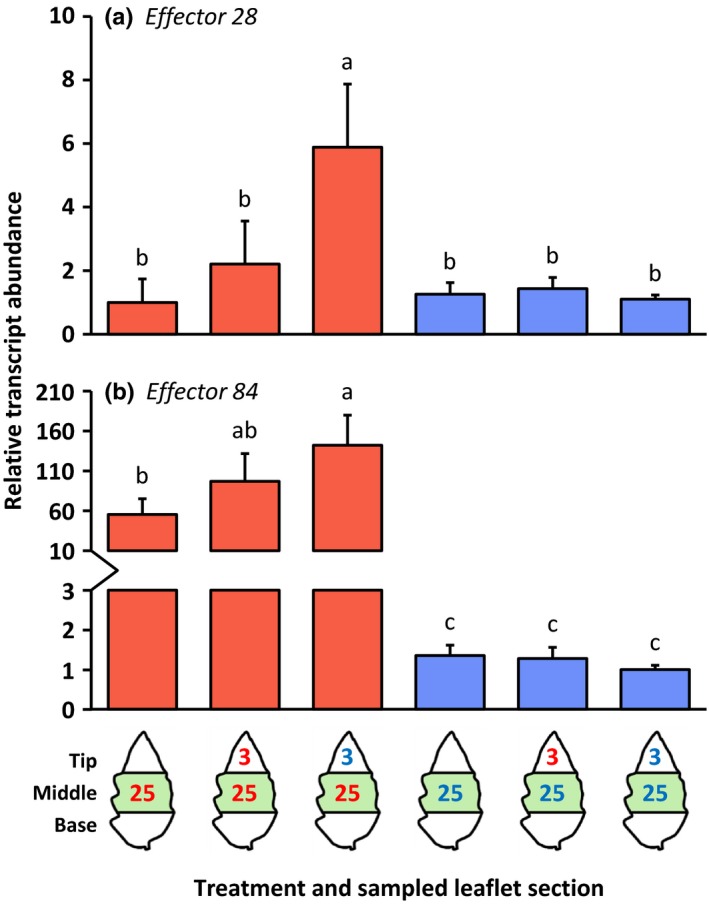
Relative transcript abundances of effector‐encoding *Tetranychus evansi* and *Tetranychus urticae* genes at the primary feeding sites of spider mite‐infested tomato (*Solanum lycopersicum*) leaflets. Using artificial barriers, leaflets of intact plants were divided into three sections: base, middle and tip. The middle section was infested with 25 *T. evansi* mites (red letters) or 25 *T. urticae* mites (blue letters), or remained uninfested as a control. After 2 d, the tip section was subjected to a secondary infestation with either three *T. evansi* mites or three *T. urticae* mites. Leaflets with uninfested tip sections were used as controls. Again 2 d later, leaflets were excised and leaflet sections were cut out for hormone extraction and RNA isolation (Supporting Information Fig. S1). The figure shows the average (+ SEM) normalized transcript abundances of (a) *effector 28*, that is, *T. evansi secreted protein 28* (*Te28*) and *T. urticae secreted protein 28* (*Tu28*); and (b) *effector 84*, that is, *T. evansi secreted protein 84* (*Te84*) and *T. urticae secreted protein 84* (*Tu84*), in the leaflet middle sections (indicated in green). Transcript abundances were normalized to *Ribosomal protein 49* and then scaled to the lowest average value per gene panel. Bars are colored according to the treatment of the middle section (primary infestation). Different letters above the bars indicate significant differences at a level of *P *≤* *0.05, after applying a linear mixed‐effects model followed by Tukey multiple comparisons.

As we found that levels of defense‐associated phytohormones as well as gene transcripts in the primary *T. evansi* feeding site were lowest after a secondary infestation with *T. urticae*, we determined the transcript abundances of two effector‐encoding *T. evansi* genes, that is, *Te28* and *Te84* (Villarroel *et al*., 2016), to assess if their expression levels correlated with the observed hyper‐suppression. The expression of *Te28* and *Te84* was significantly induced in *T. evansi* mites in response to a secondary infestation with *T. urticae* compared with when the tip remained uninfested (Fig. 6; red bars). The secondary infestation with conspecifics resulted in intermediate expression levels of *Te84*. By contrast, expression of the orthologous *Tu28* and *Tu84* (Villarroel *et al*., 2016) did not increase in *T. urticae* after a secondary infestation with mites from either of the species (Fig. 6; blue bars). Moreover, transcripts of *Te84* were significantly more abundant in *T. evansi* than transcripts of *Tu84* were in *T. urticae*, irrespective of a secondary infestation (Fig. 6b). For *Te28* versus *Tu28*, this was only the case after *T. urticae* had been introduced to the tip section.

When overexpressed in *Nicotiana benthamiana*,* Te28*,* Te84*,* Tu28* and *Tu84* each suppress SA defenses (Villarroel *et al*., 2016). To test if these effectors also affect induced JA defenses, we used *Agrobacterium tumefaciens* for the transient overexpression of the effector‐encoding genes in *N. benthamiana* leaves and assessed transcript levels of the JA‐responsive *trypsin proteinase inhibitor* (*TPI*) (Yoon *et al*., 2009) after the induction of JA defenses in agro‐infiltrated leaves by wounding and subsequent application of *Manduca sexta* oral secretions to the wounds (W + OS treatment; Methods S3). Compared with the empty vector control, the transient overexpression of *Te28*,* Te84*,* Tu28* or *Tu84* significantly reduced the transcript accumulation of *TPI* in W + OS‐treated leaves (Fig. S3). Hence, hyper‐suppression of defenses by *T. evansi* coincided with the increased transcript abundance of salivary effector genes which – in turn – suppress JA and SA defenses.

### Performance of spider mites on the primary feeding site in response to a secondary infestation of adjacent leaflet tissue

**Figure 7:**
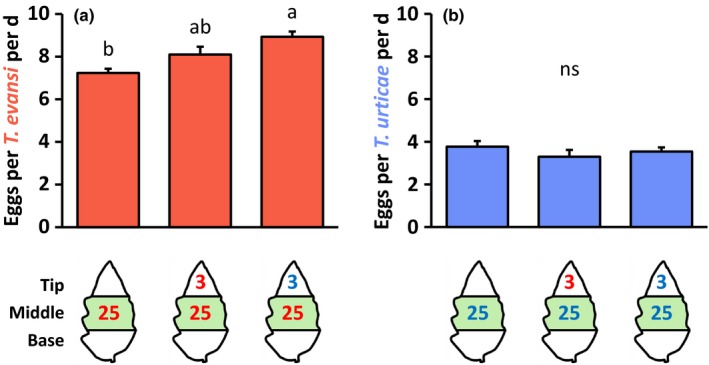
Reproductive performance of adult *Tetranychus evansi* and *Tetranychus urticae* females at the primary feeding sites of spider mite‐infested tomato (*Solanum lycopersicum*) leaflets. Using artificial barriers, leaflets of intact plants were divided into three sections: base, middle and tip. The middle section was infested with 25 *T. evansi* mites (red letters) or 25 *T. urticae* mites (blue letters). After 2 d, the tip section was subjected to a secondary infestation with either three *T. evansi* mites or three *T. urticae* mites. Leaflets with uninfested tip sections were used as controls. Again 2 d later, the number of eggs produced by the mites on the leaflet middle section (indicated in green) was counted. The figure shows the average (+ SEM) number of eggs produced per female per day for (a) *T. evansi* and (b) *T. urticae*. Bars are colored according to the treatment of the middle section (primary infestation). Different letters above the bars indicate significant differences at a level of *P *≤* *0.05, after applying a generalized linear model followed by Tukey multiple comparisons. ns, not significant.

To assess the biological significance of hyper‐suppression of tomato defenses by *T. evansi*, we analyzed the performance of the *T. evansi* females. The oviposition rate of *T. evansi* on the primary feeding site (middle section) significantly increased when the tip section was subjected to a secondary infestation with three *T. urticae* mites, whereas infestation of the tip with three conspecific mites resulted in an intermediate oviposition rate (Fig. 7a). The survival of the *T. evansi* mites on the primary feeding site was not significantly affected by the secondary infestation treatment (GLM; *F*_[2,61]_ = 1.22; *P* = 0.30). Thus, hyper‐suppression of defenses coincided with a higher oviposition rate. Similarly, we analyzed the performance of the *T. urticae* females. The oviposition rate of *T. urticae* on the leaflet middle section was not significantly affected by the secondary infestation (Fig. 7b), and neither was the survival of these mites (GLM; *F*_[2,54]_ = 2.88; *P* = 0.06).

## Discussion

Here we have shown that induction of JA and SA defenses by *T. urticae* is predominantly local, that is, restricted to their feeding site. Typically, also suppression of JA and SA defenses by *T. evansi* was observed locally. Nevertheless, a secondary infestation of the same leaflet with *T. urticae* promoted defense suppression by *T. evansi*. We found that JA and SA defenses in *T. evansi*'s feeding site were suppressed more strongly upon a secondary infestation of adjacent leaf tissue with *T. urticae*. This hyper‐suppression coincided with the increased expression of effector‐encoding *T. evansi* genes and, importantly, it was paralleled by an increased reproductive performance of *T. evansi*. These results suggest that in response to nearby competitors *T. evansi* overcompensates through the enhanced suppression of plant defenses.

### Induction of defenses by *T. urticae* and *T. evansi* is predominantly local

We found that the *T. urticae*‐induced accumulation of jasmonates, SA and transcripts of downstream marker genes was largely local (Note S1). The same was true for *T. evansi*, albeit that accumulation was at much lower absolute levels as a result of suppression (Sarmento *et al*., 2011a; Alba *et al*., 2015). In a previous study, we reported significant differences between the phytohormone profiles of *T. urticae*‐ and *T. evansi*‐infested leaflets (Alba *et al*., 2015). Here we also found such differences at the local 2‐d‐old feeding sites, but not at the 4‐d‐old ones (Figs. 1, 2). The absence/presence of significant differences in phytohormone profiles between *T. evansi*‐ and *T. urticae*‐infested leaf material might be the result of distinct infestation regimes and sampling methods that have been used, that is, 15 mites per leaflet in Alba *et al*. (2015) versus three or 25 mites per one‐third of a leaflet here. No matter the cause, the absence/presence of significant differences is not likely to be biologically relevant in these assays, as Alba *et al*. (2015) demonstrated that defense suppression by *T. evansi* takes place downstream of phytohormone accumulation. Indeed, when compared with *T. urticae*‐infested leaves, we found the expression of *PPO‐D*,* JIP‐21*,* PI‐IIc*,* PR‐1a* and *PR‐P6* to be suppressed by *T. evansi* in at least one of the leaflet sections (Figs 3, 4, 5).

The simultaneous accumulation of jasmonates and SA, concomitant with the increased expression of JA and SA marker genes at the feeding site of *T. urticae*, indicates that these two hormonal signaling pathways do not necessarily exclude each other via antagonistic crosstalk (Kant *et al*., 2004; Alba *et al*., 2015; Martel *et al*., 2015). It was suggested that the mixed response may actually reflect two spatially separated responses, because mite‐infested leaves usually contain both damaged and undamaged areas that may display different defenses (Alba *et al*., 2015). The data presented here do not support that idea and reinforce the notion that this mixed response really results from the two responses being executed at the same time, at the same place. Nonetheless, the magnitude of both responses may be intermediate as a result of JA–SA crosstalk (Glas *et al*., 2014).

### *Tetranychus urticae* evokes hyper‐suppression of defenses by *T. evansi* when infesting the same leaflet

We observed that the majority of surveyed JA and SA defenses in the local *T. evansi*‐damaged tissues were suppressed significantly more strongly when the adjacent leaf tissues were infested with defense‐inducing *T. urticae* mites (Figs 1, 2, 3, 4, 5). Furthermore, the hormones and genes that showed no significant differences in terms of hyper‐suppression exhibited the same downward trend or were already fully suppressed in *T. evansi*‐infested leaflets. Conversely, hyper‐suppression of defenses was not detected in *T. urticae*‐infested tissues upon a secondary infestation with either hetero‐ or conspecifics (nor was hyper‐induction).

We subsequently analyzed the expression of the spider mite genes *Te28*,* Te84*,* Tu28* and *Tu84*, which encode secreted salivary proteins capable of suppressing SA defenses (Villarroel *et al*., 2016) and JA defenses (Fig. S3). First, we found the expression of *Te28* and *Te84* to be up‐regulated in *T. evansi* mites in response to the secondary infestation by *T. urticae*, whereas the expression of *Tu28* and *Tu84* in *T. urticae* did not change in response to either of the secondary infestations (Fig. 6). This suggests that *T. evansi* perceives the nearby presence of (defense‐inducing) competitors and responds by elevating production of effector proteins to suppress JA and SA defenses more strongly. A similar *T. urticae*‐induced response has been found in *T. evansi* with respect to web production. *Tetranychus evansi* covers its feeding site with silken web, which hinders invading heterospecific competitors (Sarmento *et al*., 2011b). Following the perception of yet unidentified cues emanating from *T. urticae* feeding sites, *T. evansi* produced a denser web, presumably to increase exclusion of *T. urticae* (Sarmento *et al*., 2011b). Together, these data show that one or more cues from (feeding) competitors can alter *T. evansi*'s behavior.

Second, we found marked differences in the expression levels of these effector‐encoding genes between mites of the two species, with higher transcript abundances in *T. evansi* than in *T. urticae* (up to 140 times higher), in particular after introduction of heterospecifics to the adjacent leaf area (Fig. 6). Such differences may explain why mites that express similar functional effector orthologs (Villarroel *et al*., 2016) nonetheless have a distinct effect on induced plant defenses. Quantitative differences in the expression levels of effector‐encoding genes have also been observed between virulent and avirulent isolates of filamentous phytopathogens when infecting plants and, hence, are thought to determine their degree of virulence (Cooke *et al*., 2012; Hacquard *et al*., 2013). Consistently, when feeding from tomato, the transcript abundances of these (*Te28*,* Tu28*,* Te84* and *Tu84*) and (putative) other (Jonckheere *et al*., 2016) spider mite effector‐encoding genes probably affect the magnitude of key defenses and concomitantly the performance of the mites. Given that the magnitude of JA and SA defenses reported here (Figs 1, 2, 3, 4, 5) correlates well with the transcript levels of mite effector‐encoding genes (Fig. 6), as well as with mite reproductive performance (Fig. 7), the next step would be to verify their degree of causality.

### Hyper‐suppression coincides with enhanced reproductive performance of *T. evansi*

The oviposition rate of *T. evansi* increased significantly – by well over 20% – in response to the presence of *T. urticae* on adjacent tissues (Fig. 7a). Note that spider mite population growth is exponential, and hence this difference in oviposition increases exponentially in each generation cycle (2 wk under our experimental conditions). It was suggested that herbivores with a similar feeding mode generally antagonize each other when feeding simultaneously from the same plant as a result of systemically induced plant defenses (Soler *et al*., 2013). Our data, though, indicate that suppressor mites may actually be promoted by the nearby presence of (defense‐inducing) competitors with the same feeding mode. Previously, we showed that the reproductive performance of *T. urticae* mites also increases when they reside on a leaf area adjacent to a *T. evansi* feeding site for 4 d (Alba *et al*., 2015). However, during the shorter 2‐d period that *T. urticae* resided adjacent to *T. evansi* in the experiments presented here, the performance of *T. urticae* did not improve significantly (yet) (Figs 7b, S2). Nonetheless, we demonstrated that already within these 2 d the reproductive performance of *T. evansi* had improved when *T. urticae* was present. Therefore, the plant‐mediated facilitation of *T. evansi* by *T. urticae* appears to take effect before *T. urticae* may benefit from the defense suppression by *T. evansi*. The JA–SA crosstalk mechanism, which is thought to be involved in the plant‐mediated facilitation of caterpillars by aphids (Ali & Agrawal, 2014; Kroes *et al*., 2015), is probably not involved here, because our data show concurrent (as opposed to inverse) JA and SA responses in the tissue sections of co‐infested leaflets: both JA and SA responses are induced in the tip section by *T. urticae* and hyper‐suppressed in the middle section by *T. evansi*.

### Competitor‐induced overcompensation by *T. evansi*

Overcompensation is a poorly understood phenomenon characterized by an increase in productivity – that is, growth rate, biomass production and/or reproductive output – after a stress relative to unstressed control conditions (Belsky, 1986). The overcompensation of growth and reproduction after stresses, such as food deprivation, has been reported for vertebrates (Hayward *et al*., 1997; Ab Ghani & Merilä, 2015) and invertebrates (Dmitriew & Rowe, 2007). Yet, overcompensation is especially known from plants, where it is characterized by an increase in productivity after tissue damage or tissue removal (e.g. by grazing) in comparison to uninjured plants (e.g. Paige & Whitham, 1987; Lennartsson *et al*., 1997). As with animals, there is little consensus on how frequently such overcompensation responses occur and, most importantly, how they should be interpreted (Belsky *et al*., 1993; Hawkes & Sullivan, 2001; Wise & Abrahamson, 2007). It was suggested that overcompensation responses of plants have evolved as a tolerance mechanism to facilitate regrowth (Belsky *et al*., 1993; Strauss & Agrawal, 1999) or as a mechanism to aid the herbivore‐mediated recycling of a limiting nutrient (De Mazancourt *et al*., 1998). In addition, it was suggested that overcompensation may be indicative of a mutualism between plants and herbivores, that is, when a plant tolerates a herbivore and thereby can use the resources otherwise used for defenses to increase its productivity, while also allowing the herbivore to benefit from these resources (Agrawal, 2000). For most of the reports on overcompensation by plants and animals, it is unclear how often these responses are adaptive or not; if they reflect plasticity in growth and/or reproduction strategies that serves to maximize fitness under unfavorable conditions; and how often there is a fitness penalty for overcompensated seed production or oviposition.

In our view, the apparent plant‐mediated reciprocal benefits for both mite species (Alba *et al*., 2015; this study) are probably not indicative of a mutualism, because mites from both species rapidly overexploit their host (Sarmento *et al*., 2011b). Hence, any mutual benefits on co‐infested plants will be short‐lived as the shared resources will be depleted even faster. Furthermore, while for the defense‐inducing *T. urticae* it is clear that they can be facilitated by the defense suppression of *T. evansi* (Sarmento *et al*., 2011b; Alba *et al*., 2015), it is not so obvious why *T. evansi* would benefit from defense‐inducing *T. urticae*. Possibly, feeding by *T. urticae* on the leaflet tip elicits the plant to reallocate resources away from the mite's feeding site (Schultz *et al*., 2013; Zhou *et al*., 2015) and these may be intercepted by *T. evansi*, who uses them to intensify suppression and increase its oviposition rate. Alternatively, the overcompensation response may reflect a behavioral change in *T. evansi* mites that enables them to reallocate more resources to competitive population growth at the expense of other behavioral activities (Sabelis, 1985) or of life‐history traits such as longevity, as has been found for insects (Djawdan *et al*., 1996; Sisodia & Singh, 2002; Wajnberg *et al*., 2012). No tradeoff was observed between reproduction and web production in *T. urticae* (Tien *et al*., 2009), which may explain why *T. evansi* can increase both its web production (Sarmento *et al*., 2011b) and its fecundity (this study) in the presence of heterospecifics.

Our study emphasizes that mimicking a natural co‐infestation process more precisely may reveal interactions that cannot be detected when using a more simplistic assay. In earlier studies, we staged the invasion of an infested plant by heterospecific competitors by introducing either an equal number of mites from both species onto the same leaflet at the same time (Alba *et al*., 2015) or by introducing them onto the same leaf disc by first removing one species and replacing it with the other (Sarmento *et al*., 2011a,b). Using these assays, hyper‐suppression was not observed. Spider mites, however, generally disperse by wind (Kennedy & Smitley, 1985). Natural plant infestations therefore usually start with few individuals. Furthermore, it is unlikely that plants in nature are simultaneously attacked by the exact same number of competitors or, alternatively, that mites colonize a plant from which the other species has already departed, especially because mites typically overexploit their host before dispersing (Kennedy & Smitley, 1985; Li & Margolies, 1993; Glas *et al*., 2014). Taken together, these considerations suggest that the hyper‐suppression phenomenon, and the parallel increase in reproductive performance, depend on the timing of the primary and secondary infestations, on the spatial arrangement of mite feeding sites, and possibly on mite densities (or ratios) at both feeding sites. Similarly, potential benefits for caterpillars on aphid‐infested plants also depend both on the sequence of arrival of the herbivores (Soler *et al*., 2012) and on the density of the aphids (Kroes *et al*., 2015).

Besides shielding their feeding site with copious amounts of web (Sarmento *et al*., 2011b) and reproductive interference (Sato *et al*., 2014, 2016), plant‐mediated hyper‐suppression may represent an additional mechanism that enables *T. evansi* to outcompete *T. urticae*. Other herbivores that facilitate competitors by suppressing defenses on a shared host plant – especially those that cannot produce web, such as aphids (Soler *et al*., 2012), russet mites (Glas *et al*., 2014) and whiteflies (Zhang *et al*., 2009) – may have evolved similar forms of plasticity to increase competitive population growth. Such traits may ultimately decide which species within herbivore communities can develop into a pest and which cannot. Hence, it is necessary to determine how herbivores that suppress defenses limit the negative effects of plant‐mediated facilitation, not only to understand how traits that facilitate competitors can persist within natural communities, but also to assess their role in pest formation.

## Author contributions

B.C.J.S., L.M.S.A., C.A.V., R.C.S. and M.R.K. conceived and designed the experiments; B.C.J.S., L.M.S.A., R.C., C.A.V. and J.M.A. conducted the experiments; R.C.S. contributed essential equipment; B.C.J.S. and L.M.S.A. analyzed the data; B.C.J.S. and M.R.K. wrote the manuscript with input from all co‐authors.

## Supporting information

Please note: Wiley Blackwell are not responsible for the content or functionality of any Supporting Information supplied by the authors. Any queries (other than missing material) should be directed to the *New Phytologist* Central Office.

**Fig. S1** Schematic overview of the experimental procedures of the spider mite infestation assay.
**Fig. S2** Reproductive performance of adult *Tetranychus evansi* and *Tetranychus urticae* females at the secondary feeding sites of spider mite‐infested tomato leaflets.
**Fig. S3** Spider mite effectors suppress the expression of the JA‐regulated and defense‐associated *trypsin proteinase inhibitor* (*TPI*) gene in *Nicotiana benthamiana* leaves.
**Table S1** Specification of the number of plants used in each experiment
**Table S2** qRT‐PCR primer specifications
**Methods S1** Isolation of phytohormones and analysis by means of LC‐MS/MS (detailed description).
**Methods S2** Gene‐expression analysis by means of qRT‐PCR (detailed description).
**Methods S3** Suppression of JA defenses by spider mite effectors.
**Notes S1** Within‐leaflet systemic effects on induced plant responses upon the *Tetranychus urticae* infestation.
