# Supplementary material for: Overcompensation of herbivore reproduction through hyper‐suppression of plant defenses in response to competition
Source: New Phytol. 2017 Apr 7;214(4):1688–701. doi: 10.1111/nph.14543 (PMC5434804; doi:10.1111/nph.14543)
Supplement: Supplementary file 1 — Fig. S1 Schematic overview of the experimental procedures of the spider mite infestation assay. Fig. S2 Reproductive performance of adult Tetranychus evansi and Tetranychus urticae females at the secondary feeding sites of spider mite‐infested tomato leaflets. Fig. S3 Spider mite effectors suppress the expression of the JA‐regulated and defense‐associated trypsin proteinase inhibitor (TPI) gene in Nicotiana benthamiana leaves. Table S1 Specification of the number of plants used in each experiment Table S2 qRT‐PCR primer specifications Methods S1 Isolation of phytohormones and analysis by means of LC‐MS/MS (detailed description). Methods S2 Gene‐expression analysis by means of qRT‐PCR (detailed description). Methods S3 Suppression of JA defenses by spider mite effectors. Notes S1 Within‐leaflet systemic effects on induced plant responses upon the Tetranychus urticae infestation. [file NPH-214-1688-s001.pdf]

New Phytologist

New Phytologist Supporting Information: Figs S1–S3, Tables S1 and S2, Methods S1–S3 and Notes S1

Article title: Overcompensation of herbivore reproduction through hyper-suppression of plant defenses in response to competition

Authors: Bernardus C. J. Schimmel, Livia M. S. Ataide, Rachid Chafi, Carlos A. Villarroel, Juan M. Alba, Robert C. Schuurink and Merijn R. Kant

Article acceptance date: 14 February 2017

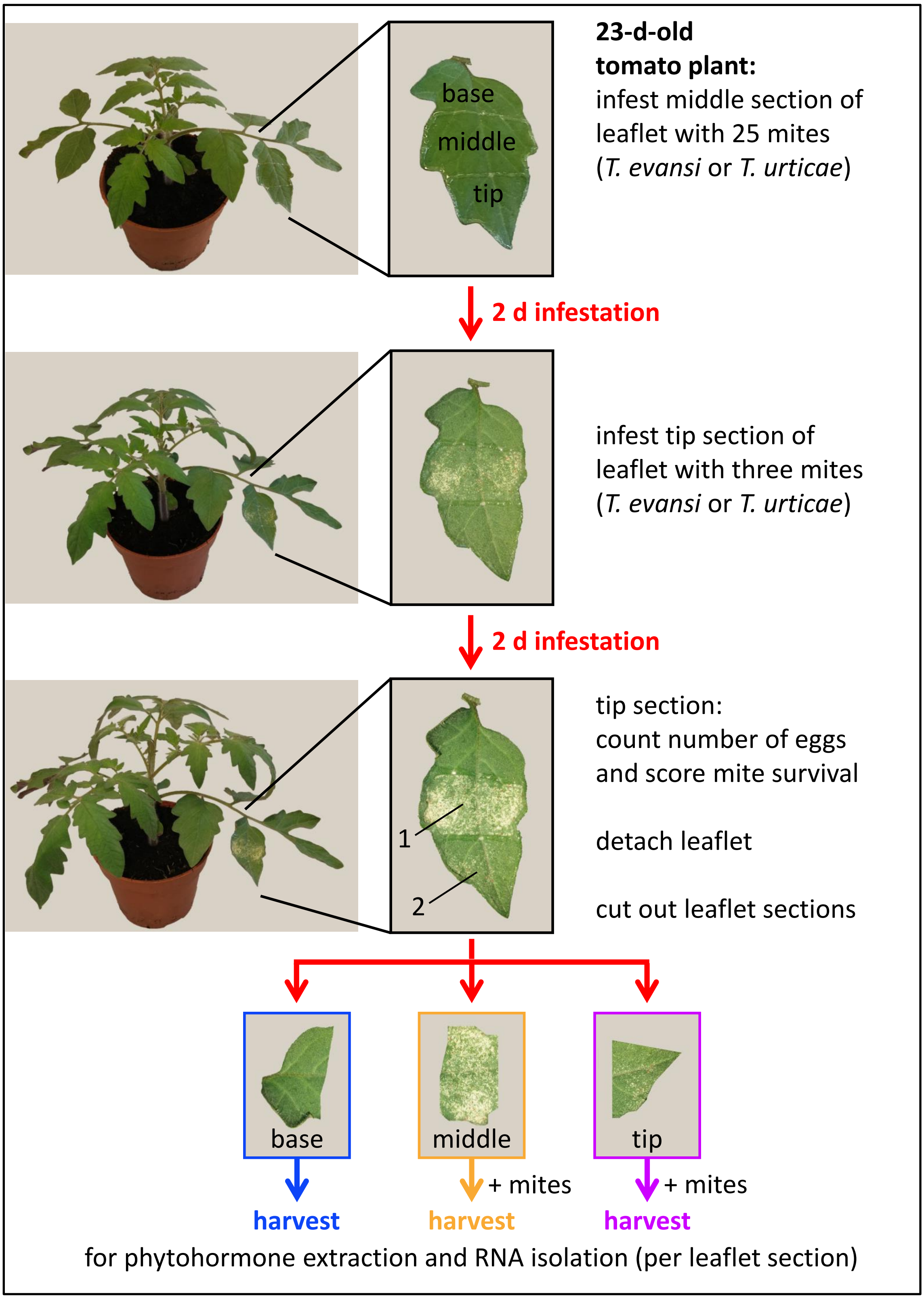

Fig. S1 Schematic overview of the experimental procedures of the spider mite infestation assay to assess the induction of plant defenses at a 4-d-old primary spider mite feeding site and in adjacent leaflet tissues including a 2-d-old secondary feeding site. For a detailed description we refer to the material and methods section. 1 = 4-d-old primary feeding site; 2 = 2-d-old secondary feeding site.

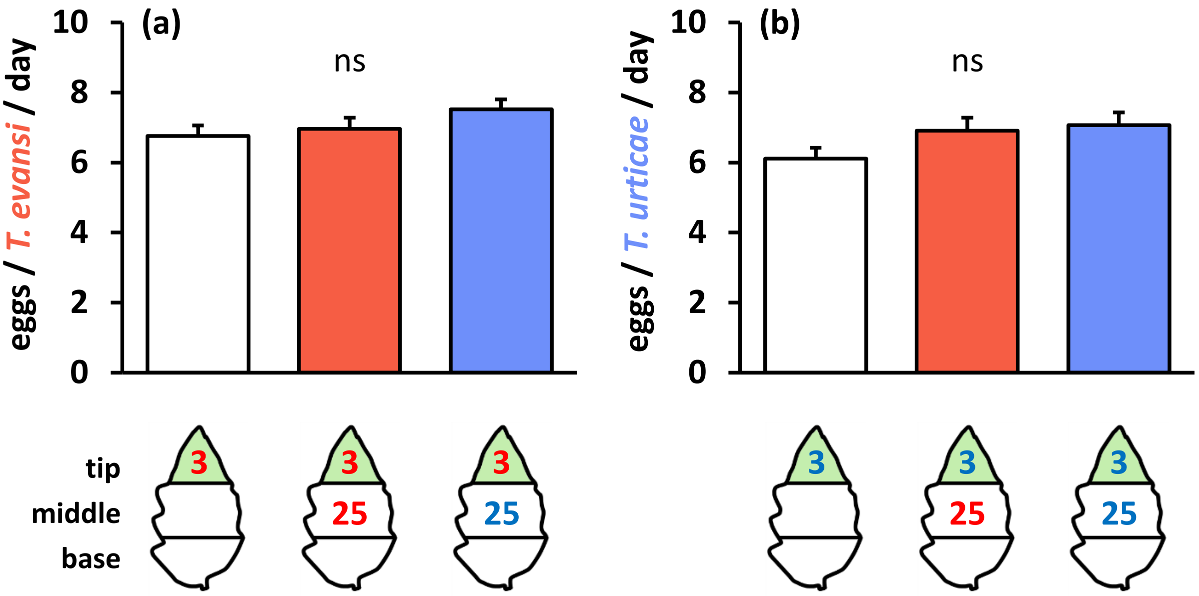

Fig. S2 Reproductive performance of adult *Tetranychus evansi* and *T. urticae* females at the secondary feeding sites of spider mite-infested tomato (*Solanum lycopersicum*) leaflets. Using artificial barriers leaflets of intact plants were divided into three sections: base, middle, tip. The middle section was infested with 25 *T. evansi* (red letters), 25 *T. urticae* (blue letters), or remained uninfested as a control. After 2 d the tip section was subjected to a secondary infestation with either three *T. evansi* or three *T. urticae*. Again 2 d later the number of eggs produced by the mites on the leaflet tip section (indicated in green) was counted. The figure shows the average (+ SEM) number of eggs produced per female per day for (a) *T. evansi* and (b) *T. urticae*. Bars are colored according to the treatment of the middle section (primary infestation). Oviposition data was statistically evaluated per mite species by applying a linear mixed-effects model, but no significant differences were found between the treatments ($P > 0.05$). ns, not significant.

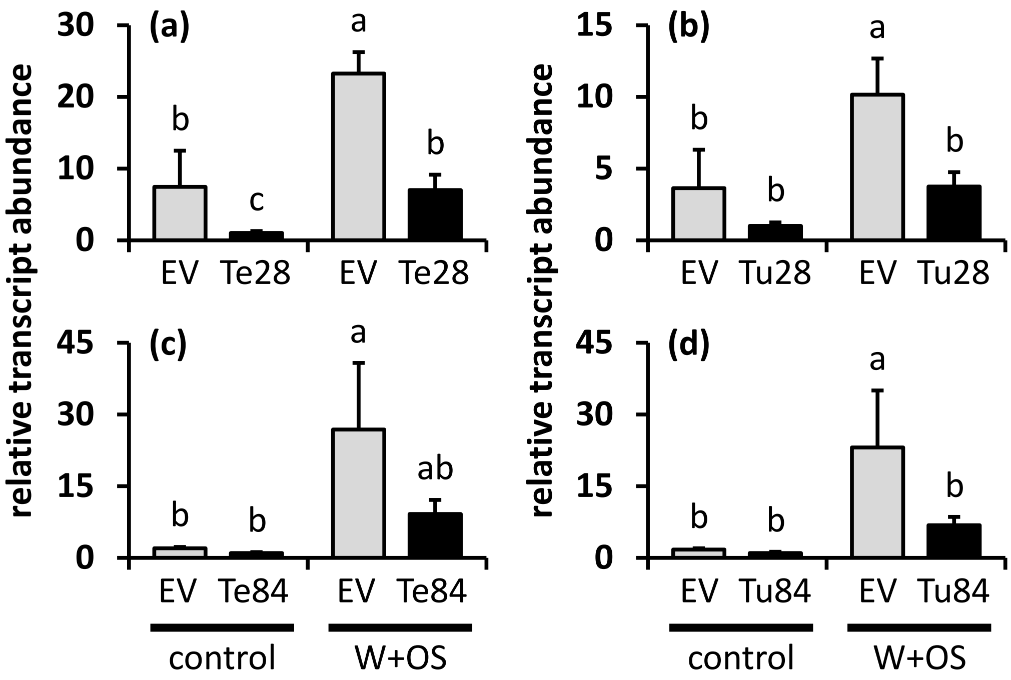

Fig. S3 Spider mite effectors suppress the expression of the jasmonic acid-regulated and defense-associated *trypsin proteinase inhibitor* (*TPI*) gene in *Nicotiana benthamiana* leaves. The figure shows the average (+ SEM) normalized transcript abundances of *TPI* 2 d after agroinfiltration with constructs containing (a) *Te28*, (b) *Tu28*, (c) *Te84*, and (d) *Tu84*, each fused downstream of the CaMV 35S promoter, or after agroinfiltration with the empty vector (EV) construct. To visualize suppression of jasmonic acid-regulated defense responses, agroinfiltrated leaves were wounded with a pattern wheel, after which *Manduca sexta* oral secretions were applied to the wounds (W+OS treatment). Untreated plants served as controls. *TPI* transcript abundances were normalized to *Actin* and then scaled to the lowest average value per treatment. Different letters above the bars indicate significant differences at a level of $P \leq 0.05$, after applying a linear mixed-effects model (panels a, b) or a generalized linear model (panels c, d), followed by Tukey multiple comparisons. For a detailed description of the experimental procedures we refer to Methods S3.

Table S1 Specification of the number of plants used in each experiment

treatment (per leaflet section)			sampled leaflet section	number of plants per treatment		
base	middle[1]	tip[2]		hormones, gene expr. and mite performance[3]	*T. evansi* performance[4]	*T. urticae* performance[5]
uninfested	uninfested	uninfested	base	45 - 40		
uninfested	uninfested	3 *T. evansi*	base	45 - 40		
uninfested	uninfested	3 *T. urticae*	base	56 - 50		
uninfested	25 *T. evansi*	uninfested	base	45 - 40		
uninfested	25 *T. evansi*	3 *T. evansi*	base	56 - 50		
uninfested	25 *T. evansi*	3 *T. urticae*	base	56 - 50		
uninfested	25 *T. urticae*	uninfested	base	56 - 50		
uninfested	25 *T. urticae*	3 *T. evansi*	base	45 - 40		
uninfested	25 *T. urticae*	3 *T. urticae*	base	45 - 40		
uninfested	uninfested	uninfested	middle	45 - 40		
uninfested	uninfested	3 *T. evansi*	middle	45 - 40		
uninfested	uninfested	3 *T. urticae*	middle	56 - 50		
uninfested	25 *T. evansi*	uninfested	middle	45 - 40	22	
uninfested	25 *T. evansi*	3 *T. evansi*	middle	56 - 50	20	
uninfested	25 *T. evansi*	3 *T. urticae*	middle	56 - 50	23	
uninfested	25 *T. urticae*	uninfested	middle	56 - 50		19
uninfested	25 *T. urticae*	3 *T. evansi*	middle	45 - 40		19
uninfested	25 *T. urticae*	3 *T. urticae*	middle	45 - 40		19
uninfested	uninfested	uninfested	tip	45 - 40		
uninfested	uninfested	3 *T. evansi*	tip	45 - 40 - 45		
uninfested	uninfested	3 *T. urticae*	tip	56 - 50 - 56		
uninfested	25 *T. evansi*	uninfested	tip	45 - 40		
uninfested	25 *T. evansi*	3 *T. evansi*	tip	56 - 50 - 54		
uninfested	25 *T. evansi*	3 *T. urticae*	tip	56 - 50 - 52		
uninfested	25 *T. urticae*	uninfested	tip	56 - 50		
uninfested	25 *T. urticae*	3 *T. evansi*	tip	45 - 40 - 42		
uninfested	25 *T. urticae*	3 *T. urticae*	tip	45 - 40 - 41		

1, primary infestation

2, secondary infestation

3, numbers indicate (from left to right): total number of plants per treatment - number of plants harvested for phytohormone and RNA isolation - number of replicates used to determine the reproductive performance of the mites at the tip section (either *T. evansi* or *T. urticae*). Rows are colored according to the treatment of the leaflet middle section (primary infestation). The phytohormone data is presented in Figs 1, 2; gene expression data is presented in Figs 3, 4, 5, 6; mite reproductive performance data (secondary infestation) is presented in Fig. S2. Note that 10 leaflet sections (i.e., either base, middle or tip) obtained from 10 plants were pooled to form one biological replicate to have a sufficient amount of leaf material to enable phytohormone extraction and RNA isolation from the same sample. Hence, 40 plants results in four biological replicates and 50 plants in five biological replicates.

4, *T. evansi* reproductive performance data (primary infestation) is presented in Fig. 7(a).

5, *T. urticae* reproductive performance data (primary infestation) is presented in Fig. 7(b).

New Phytologist

Table S2 qRT-PCR primer specifications

Target Organism(s)	Target Gene	Name	Gene Identifier	Forward Primer 5' → 3'	Reverse Primer 5' → 3'	References
Solanum lycopersicum	*OPR3*	*OPDA reductase 3*	Solyc07g007870.2	GATCCAGTTGTGGGATACACAG	GCCCAACAAAATCAGGTTTC	Strassner *et al*. (2002)
Solanum lycopersicum	*PPO-D*	*Polyphenol-oxidase-D*	Solyc08g074680.2	GCCCAATGGAGCCATATC	ACATTCGATCCACATTGCTG	Newman *et al*. (1993)
Solanum lycopersicum	*JIP-21*	*Jasmonate-inducible protein 21*	Solyc03g098790.1	ACTCGTCCTGTGCTTTGTCC	CCCAAGAGGATTTTCGTTGA	Lisón *et al*. (2006)
Solanum lycopersicum	*PI-IIc*	*Proteinase Inhibitor IIc*	Solyc03g020050.2	CAGGATGTACGACGTGTTGC	GAGTTTGCAACCCTCTCCTG	Gadea *et al*. (1996)
Solanum lycopersicum	*PR-1a*	*Pathogenesis-related protein 1a*	Solyc09g007010.1	TGGTGGTTCATTTCTTGCAACTAC	ATCAATCCGATCCACTTATCATTTTA	Van Kan *et al*. (1992)
Solanum lycopersicum	*PR-P6*	*Pathogenesis-related protein P6*	Solyc00g174340.1	GTACTGCATCTTCTTGTTTCCA	TAGATAAGTGCTTGATGTGCC	Van Kan *et al*. (1992)
Solanum lycopersicum	*Actin*	*Actin*	Solyc03g078400.2	TCAGCACATTCCAGCAGATGT	AACAGACAGGACACTCGCACT	Tomato Genome Consortium (2012)
Nicotiana benthamiana	*TPI*	*Trypsin proteinase inhibitor*	DQ158182.1	ACTTTCGAATGCGATCCAAG	TCAACCACTTTGCTGCCATA	Villarroel *et al*. (2016)
Nicotiana benthamiana	*Actin*	*Actin*	JQ256516.1	CGGAATCCACGAGACTACATAC	GGGAAGCCAAGATAGAGC	Villarroel *et al*. (2016)
Tetranychus evansi	*Te28*	*Tetranychus evansi secreted protein 28*	KT182959	CCAAGCACAACGCTGAAGA	ATTGGCTGGAAACTGATTGG	Villarroel *et al*. (2016)
Tetranychus evansi	*Te84*	*Tetranychus evansi secreted protein 84*	KT182961	AACAAATGATTGGTGGCCTTG	TTCGAACAATTTACCGGATGC	Villarroel *et al*. (2016)
Tetranychus urticae	*Tu28*	*Tetranychus urticae secreted protein 28*	tetur31g01040	AGTTCTCTGACGAAGCCAAG	GCTGTAGCGATATCAGCAAG	Villarroel *et al*. (2016)
Tetranychus urticae	*Tu84*	*Tetranychus urticae secreted protein 84*	tetur01g01000	TCTCAGTTGGTGGTGCTTTC	CGTTCATGGCATTGTCAAGG	Villarroel *et al*. (2016)
Tetranychus evansi & *Tetranychus urticae*	*RP49*[1]	*Ribosomal protein 49*	tetur18g03590 Tevan_rep_c13981	CTTCAAGCGGCATCAGAGC	CGCATCTGACCCTTGAACTTC	Villarroel *et al*. (2016)

1, the *RP49* amplicon is identical for both mite species

Methods S1

Isolation of phytohormones and analysis by means of LC-MS/MS

About 150-300 mg of frozen leaf material was homogenized (Precellys 24, Bertin Technologies, Aix-en-Provence, France) in 1 ml of ethyl acetate. The ethyl acetate had been spiked with D_6-SA and D_5-JA (C/D/N Isotopes Inc, Canada) as internal standards with a final concentration of 100 ng ml^{-1}. Tubes were centrifuged at 13,000 rpm (15,493 ***g***; Sigma 3-30KS; SIGMA Laborzentrifugen GmbH, Osterode am Harz, Germany) for 10 min at 4°C and the supernatant (the ethyl acetate phase) was transferred to new tubes. The pellet was re-extracted with 0.5 ml of ethyl acetate (without internal standards) and centrifuged again at 13,000 rpm for 10 min at 4°C. Both supernatants were combined and evaporated to dryness on a vacuum concentrator (CentriVap Centrifugal Concentrator, Labconco, Kansas City, MO, USA) at 30°C. The residue was re-suspended in 0.1 ml 70% methanol (v/v), centrifuged at 14,800 rpm (20,081 ***g***) for 15 min at 4°C, and the supernatants were transferred to glass vials and then analyzed by means of LC-MS/MS. A serial dilution of pure standards of OPDA, JA, JA-Ile and SA was run separately. Measurements were conducted on a liquid chromatography tandem mass spectrometry system (Varian 320-MS LC/MS, Agilent Technologies). We injected 20 µl of each sample onto a Kinetix 5u C18 100A column (C18 phase, 5 µm particle size, 100Å pore size, 50 × 2.1 mm, Phenomenex, Torrance, CA, USA) equipped with a Phenex-RC guard cartridge (Phenomenex). The mobile phase contained solvent A (0.05% formic acid in LCMS-grade water; Sigma-Aldrich, St. Louis, MO, USA) and solvent B (0.05% formic acid in LCMS-grade methanol; Sigma-Aldrich) in varying proportions. The program, with a constant flow rate of 0.2 ml min^{-1}, was set as follows: (i) 95% solvent A/5% solvent B for 1 min 30 sec; (ii) followed by 6 min in which solvent B gradually increased till 98%; (iii) continuing with 98% solvent B for 5 min; (iv) then a rapid (in 1 min) but gradual decrease returning to 95% solvent A/5% solvent B until the end of the run. A negative electrospray ionization mode was used for detection. LC-MS/MS parameters, i.e., the parent ions, daughter ions, and collision energies were identical to those of Alba *et al.* (2015). For all oxylipins we used D_5-JA to estimate the recovery rate and their *in planta* concentrations were subsequently quantified using the respective external standard series. For SA we used D_6-SA to estimate the recovery rate and it was quantified using the external standard series.

Methods S2

Gene-expression analysis by means of quantitative reverse-transcriptase PCR (qRT-PCR)

Total RNA was isolated from tomato tissue (with or without mites) using the hot phenol method (Verwoerd *et al.*, 1989). The NanoDrop spectrophotometer (ND-1000, Thermo Fisher Scientific, Waltham, MA, USA) was used to assess RNA purity and quantity. DNase (Ambion, Austin, TX, USA)-treated RNA was used as template for reverse transcription and first strand cDNA synthesis using RevertAid H Minus Reverse Transcriptase (Thermo Fisher Scientific). For gene expression analysis, 1 µl of diluted cDNA (i.e. the equivalent of 7.5 ng total RNA for tomato genes and 100 ng total RNA for spider mite genes) served as template in a 20 µl qRT-PCR using the 5x HOT FIREPol EvaGreen qPCR Mix Plus (ROX) kit (Solis Biodyne, Tartu, Estonia) and the ABI 7500 Real-Time PCR system (Applied Biosystems, Foster City, CA, USA), according to the instructions of the manufacturers. Using RNA from the same samples, we analyzed the transcript abundance of the tomato defense-associated marker genes *OPR3*, *PPO-D*, *JIP-21*, *PI-IIc*, *PR-1a* and *PR-P6*, as well as the spider mite effector-encoding genes *Te28*, *Tu28*, *Te84* and *Tu84*. With the exception of *OPR3*, the expression patterns of the tomato genes over time in plants infested with mites from the *T. urticae* or *T. evansi* lines used here have been described in detail before (Alba *et al.*, 2015). Tomato *Actin* and spider mite *RP49* were used as

reference genes for the respective template to normalize expression data across samples. Gene identifiers, primer sequences and references are listed in Table S2. Primer efficiency of each primer pair was calculated using standard dilution series. qRT-PCR-generated amplicons were sequenced to verify primer specificity. When gene transcripts were not detected in both technical replicates of a sample, then this sample was scored as ‘0’ (zero) and included as such in the statistical analysis. When gene transcripts were detected in only one technical replicate of one biological replicate and not in all other replicates of the same treatment, then the normalized expression (NE) of that one technical replicate was also scored as ‘0’. The NE data were calculated by the ΔCt method: NE = $(PE_{target}^{Ct\_target})/(PE_{reference}^{Ct\_reference})$; in which PE is the primer efficiency and Ct the number of cycles to reach the cycle threshold value. To plot the relative expression, NE values were scaled to the treatment with the lowest average NE.

Methods S3

Suppression of JA defenses by spider mite effectors

Previously, using the *Agrobacterium tumefaciens*-mediated transient overexpression of spider mite effector-encoding genes in *Nicotiana benthamiana* leaves, Te28, Tu28, Te84 and Tu84 have been shown to suppress SA defenses (Villarroel *et al.*, 2016). Although there were indications that (some of) these effectors also suppressed JA defenses, the agroinfiltration-induced SA response and concomitant antagonistic crosstalk with the JA pathway largely concealed the experimental outcome with respect to suppression of JA defenses (Villarroel *et al.*, 2016). Hence, here we deliberately induced JA-regulated defense responses in agroinfiltrated *N. benthamiana* leaves to be able to detect suppression of these defenses by spider mite effectors. Cloning of each of the four mite effector-encoding genes (without signal peptide) into the plant expression vector pSOL2092, which contains the *Cauliflower mosaic virus* 35S promoter, and subsequent *A. tumefaciens*-mediated transient overexpression assays with mite effector or empty vector (EV) constructs in *N. benthamiana* were performed as described by Villarroel *et al.* (2016). Two days after agroinfiltration, leaves were wounded with a pattern wheel, after which 20 µl of *Manduca sexta* oral secretions (three times diluted) was applied to the wounds (W+OS treatment) as described by Wu *et al.* (2007). Four hours after the W+OS treatment the leaves were harvested for RNA isolation. Control plants were agroinfiltrated but did not receive the W+OS treatment. RNA isolation, DNAse-treatment, cDNA synthesis and qRT-PCRs to determine the transcript abundance of the JA-responsive *N. benthamiana trypsin proteinase inhibitor* (*TPI*) gene (Yoon *et al.*, 2009) were performed as described in Methods S2. *TPI* transcript abundances were normalized to *Actin* (Table S2) and then scaled to the lowest average value per treatment. The NE of *TPI* was statistically evaluated per construct as described in the material and methods section. For effectors Te28 and Tu28, six plants were agroinfiltrated per treatment, while for effectors Te84 and Tu84, three plants were agroinfiltrated per treatment.

Notes S1

Within-leaflet systemic effects on induced plant responses upon the *T. urticae* infestation

From our set of marker genes, only the expression of *JIP-21*, which encodes a JA-inducible proteinase inhibitor that inhibits digestive enzymes in the gut of herbivores (Lisón *et al.*, 2006), was up-regulated locally and systemically by *T. urticae*. This is in line with results from an earlier study that showed an increased proteinase inhibitor activity in *T. urticae*-infested leaves as well as in uninfested leaves of the same plants (Sarmento *et al.*, 2011). In contrast to *JIP-21*, the expression of the JA-biosynthesis gene *OPR3* was down-regulated in tissues adjacent to the 4-d-old *T. urticae* feeding site. Locally, *OPR3*

expression was not significantly altered by *T. urticae*, yet it was induced by *T. evansi*. As noted previously, not all defense-associated plant genes are (continuously) induced by *T. urticae* and suppressed by *T. evansi*, respectively, throughout the course of the infestation (Alba *et al.*, 2015). Also the expression of *OPR3* seems to depend on the duration of the infestation and/or the intensity of the infestation, as its expression was induced in the leaflet tip section after infestation with *T. urticae* for 2 d (Fig. 3a). The RNAi-mediated silencing of *OPR3* in tomato has been shown to result in lower concentrations of jasmonates (including JA-Ile) that are produced from OPDA (Bosch *et al.*, 2014; Scalschi *et al.*, 2015). However, we found JA and JA-Ile concentrations not to significantly differ between tissues infested by *T. urticae* or *T. evansi*, nor in the adjacent tissues. This emphasizes that expression levels of phytohormone biosynthesis genes are not always predictive of the actual phytohormone concentrations, which in this case might be the result of feedback and feedforward mechanisms associated with JA biosynthesis (Wasternack & Hause, 2013).

References

Alba JM, Schimmel BCJ, Glas JJ, Ataide LM, Pappas ML, Villarroel CA, Schuurink RC, Sabelis MW, Kant MR. 2015. Spider mites suppress tomato defenses downstream of jasmonate and salicylate independently of hormonal crosstalk. *New Phytologist* **205**: 828-840.

Bosch M, Wright LP, Gershenzon J, Wasternack C, Hause B, Schaller A, Stintzi A. 2014. Jasmonic acid and its precursor 12-oxophytodienoic acid control different aspects of constitutive and induced herbivore defenses in tomato. *Plant Physiology* **166**: 396-410.

Gadea J, Mayda ME, Conejero V, Vera P. 1996. Characterization of defense-related genes ectopically expressed in viroid-infected tomato plants. *MPMI-Molecular Plant Microbe Interactions* **9**: 409-415.

Lisón P, Rodrigo I, Conejero V. 2006. A novel function for the cathepsin D inhibitor in tomato. *Plant Physiology* **142**: 1329-1339.

Newman SM, Eannetta NT, Yu H, Prince JP, de Vicente MC, Tanksley SD, Steffens JC. 1993. Organisation of the tomato polyphenol oxidase gene family. *Plant Molecular Biology* **21**: 1035-1051.

Sarmento RA, Lemos F, Bleeker PM, Schuurink RC, Pallini A, Oliveira MG, Lima ER, Kant M, Sabelis MW, Janssen A. 2011. A herbivore that manipulates plant defence. *Ecology Letters* **14**: 229-236.

Scalschi L, Sanmartin M, Camanes G, Troncho P, Sanchez-Serrano JJ, Garcia-Agustin P, Vicedo B. 2015. Silencing of OPR3 in tomato reveals the role of OPDA in callose deposition during the activation of defense responses against *Botrytis cinerea*. *Plant Journal* **81**: 304-315.

Strassner J, Schaller F, Frick UB, Howe GA, Weiler EW, Amrhein N, Macheroux P, Schaller A. 2002. Characterization and cDNA-microarray expression analysis of 12-oxophytodienoate reductases reveals differential roles for octadecanoid biosynthesis in the local versus the systemic wound response. *The Plant Journal* **32**: 585-601.

Tomato Genome Consortium T. 2012. The tomato genome sequence provides insights into fleshy fruit evolution. *Nature* **485**: 635-641.

Van Kan JA, Joosten MH, Wagemakers CA, van den Berg-Velthuis GC, de Wit PJ. 1992. Differential accumulation of mRNAs encoding extracellular and intracellular PR proteins in tomato induced by virulent and avirulent races of *Cladosporium fulvum*. *Plant Molecular Biology* **20**: 513-527.

Verwoerd TC, Dekker B, Hoekema A. 1989. A small-scale procedure for the rapid isolation of plant RNAs. *Nucleic Acids Research* **17**: 2362.

Villarroel CA, Jonckheere W, Alba JM, Glas JJ, Dermauw W, Haring MA, Van Leeuwen T, Schuurink RC, Kant MR. 2016. Salivary proteins of spider mites suppress defenses in *Nicotiana benthamiana* and promote mite reproduction. *The Plant Journal* **86**: 119-131.

Wasternack C, Hause B. 2013. Jasmonates: biosynthesis, perception, signal transduction and action in plant stress response, growth and development. An update to the 2007 review in Annals of Botany. *Annals of Botany* **111**: 1021-1058.

Wu J, Hettenhausen C, Meldau S, Baldwin IT. 2007. Herbivory rapidly activates MAPK signaling in attacked and unattacked leaf regions but not between leaves of *Nicotiana attenuata*. *Plant Cell* **19**: 1096-1122.

Yoon J, Chung WI, Choi D. 2009. NbHB1, *Nicotiana benthamiana* homeobox 1, is a jasmonic acid-dependent positive regulator of pathogen-induced plant cell death. *New Phytologist* **184**: 71-84.
